# Tin and Gold Combined

**Published:** 1886-02

**Authors:** J. H. Spalding

**Affiliations:** Lecturer on Medical and Surgical Dentistry in the Minnesota Hospital College


### TIN AND GOLD COMBINED.

BY J. H. SPALDING, D. D. S.

LECTURER ON MEDICAL AND SURGICAL DENTISTRY IN THE MINNESOTA HOSPITAL COLLEGE.

I am very much pleased to see that so representative a man in our profession in America as Dr. A. W. Harlan, of Chicago, has joined the ranks of those who have learned the value of tin and gold combined, as a filling material, and has become its advocate.

From time to time articles have appeared in the dental journals from such men as Abbott, Jenkins, Miller, Sachs, and others, of Germany, setting forth the advantages possessed by this combination for filling teeth, giving their experience in its use and recommending its many virtues, but little heed has been given by practitioners in this country, because, like the Pharisees of old, they thought no good thing could possibly come out of this Nazareth. Whatever was not distinctively American in dentistry has been deemed of no value, and has merited no consideration. For the past year I have been fighting the battles of tin and gold in this region, and have not as yet succeeded in stirring up even a spirit of investigation among my colleagues, as to its merits. I think no one has tried it. From our English ancestors we have inherited so much of that insular prejudice, the very name of which is derived from the British islands, that whatever is mentioned as having any extensive use or advocacy abroad, is looked upon as unworthy the attention of an American dentist, even in Minnesota. The glory of American dentistry is indeed great, but we must not let our pride in our achievements blind us to whatever is good, even though it emanate from another country. While in Germany, two years ago, I learned to use this combination of metals, and had abundant opportunity, during my year of practice there, to see fillings of this material, and I am free to say that I was so convinced of its immense value as a means of saving a large class of teeth, that I have used it ever since, mostly to the exclusion of amalgam, except in the most out of the way and inaccessible cavities.

It possesses all the qualities mentioned by Dr. Harlan in his article in the October number of this journal, and in addition I will mention that it slightly expands; and by reason of some change (molecular or chemical?) taking place shortly after insertion, it becomes almost as hard as the densest amalgam. This latter quality appears to be more strongly manifested when it is inserted under moisture. This is the secret of Dr. Harlan's remark, that "it is capable of resisting mastication in an astonishing manner," for these fillings have all the soft and yielding qualities of tin alone, when first completed. These two qualities, together with its ease and rapidity of insertion and freedom from injury by moisture, render this combination one of our most valuable adjuncts in the saving of teeth. The instructions given by Dr. Harlan, as to its preparation and use, are comprehensive and clear, the only objection being that he mentions using instruments mostly "wedge-shaped or three-cornered." I have found that I can do better work with these metals by having instruments entirely devoid of corners. Mine are blunt, wedge-shaped, rather large-size points, with longitudinal serrations, having no corners to tear the ribbons or ropes.

There are other materials in use in Europe, which are of European manufacture, and which American practitioners could adopt and make use of without detracting from the dignity of American dentistry. I speak of the copper amalgam (known also as Sullivan's cement), and of Poulson's or Rostaing's cements. Where it is necessary to use amalgam at all, this common copper amalgam is incomparably superior to all the mis-named "Gold-and Platina Alloys," and "non-discolorable" trash of the American market. If we are to use amalgam, let us do so for a therapeutical purpose, not as a mere mechanical stopping of a hole, for we have other materials at hand which are mechanically superior, and in all respects quite equal as to cheapness and rapidity of insertion.

Regarding the cements spoken of, my own experience and observation confirm the statements of all Americans in practice in Europe, with whom I have conversed, that there is no cement of American manufacture that at all approaches either Poulson's Mineral Plombe or Rostaing's Cement, for all uses where cements are admissible. They disintegrate less at the cervical margins, are denser and more firmly adherent to the walls of cavities, than any other cements with which I am familiar.

"Prove all things; hold fast to that which is good," is useful advice for the dentists of America to follow, and whether the thing to be proven be American or foreign, we should not be prejudiced against it. I cannot close without again reverting to my subject, Tin and Gold Combined, and urging a trial of its merits, feeling confident that it will be found to possess qualities eminently superior to those of any other material now in use for many classes of fillings.

NOTE.—Dr. Spalding is a positive man and speaks positively, but we do not think that he desires to be understood as utterly condemning all of the cements and amalgams of American manufacture. Some of these are very good, though there are many that are worthless. Sullivan's copper amalgam possesses good qualities, but all Europe, even, is not convinced of its superlative excellence.—EDITOR.

